# Built Environment, Selected Risk Factors and Major Cardiovascular Disease Outcomes: A Systematic Review

**DOI:** 10.1371/journal.pone.0166846

**Published:** 2016-11-23

**Authors:** Pasmore Malambo, Andre P. Kengne, Anniza De Villiers, Estelle V. Lambert, Thandi Puoane

**Affiliations:** 1 University of Western Cape, School of Public Health, Robert Sobukwe Rd, Bellville, Cape Town, 7535, South Africa; 2 Non-communicable disease Unit, South African Medical Research Council, Francie van Zijl Drive, Parowvallei, P.O. Box 19070, 7505 Tygerberg, Cape Town, South Africa; 3 Division of Exercise Science and Sports Medicine, Department of Human Biology, Faculty of Health Sciences, University of Cape Town, Boundary Road, Newlands, 7700, Cape Town, South Africa; University of Bologna, ITALY

## Abstract

**Introduction:**

Built environment attributes have been linked to cardiovascular disease (CVD) risk. Therefore, identifying built environment attributes that are associated with CVD risk is relevant for facilitating effective public health interventions.

**Objective:**

To conduct a systematic review of literature to examine the influence of built environmental attributes on CVD risks.

**Data Source:**

Multiple database searches including Science direct, CINAHL, Masterfile Premier, EBSCO and manual scan of reference lists were conducted.

**Inclusion Criteria:**

Studies published in English between 2005 and April 2015 were included if they assessed one or more of the neighborhood environmental attributes in relation with any major CVD outcomes and selected risk factors among adults.

**Data Extraction:**

Author(s), country/city, sex, age, sample size, study design, tool used to measure neighborhood environment, exposure and outcome assessments and associations were extracted from eligible studies.

**Results:**

Eighteen studies met the inclusion criteria. Most studies used both cross-sectional design and Geographic Information System (GIS) to assess the neighborhood environmental attributes. Neighborhood environmental attributes were significantly associated with CVD risk and CVD outcomes in the expected direction. Residential density, safety from traffic, recreation facilities, street connectivity and high walkable environment were associated with physical activity. High walkable environment, fast food restaurants, supermarket/grocery stores were associated with blood pressure, body mass index, diabetes mellitus and metabolic syndrome. High density traffic, road proximity and fast food restaurants were associated with CVDs outcomes.

**Conclusion:**

This study confirms the relationship between neighborhood environment attributes and CVDs and risk factors. Prevention programs should account for neighborhood environmental attributes in the communities where people live.

## Background

Current global mortality rates from non-communicable diseases (NCDs) remain unacceptably high and are increasing [1]. More than 70% of global cardiovascular disease (CVD), are attributable to modifiable risk factors [2]. Rapidly globalization is accompanied by increasing urbanization, population growth and changes in demographics and promotes trends towards unhealthy lifestyles [3]. The ecological model, however, states that an individual’s behaviour is influenced by multiple level factors such as social, neighborhood environment, and policy factors [4,5]. One of these factors, the neighborhood environment, and its link to health have been the focus of an increasing number of studies in recent years [6]. These studies are from a variety of disciplines, including urban planning and transportation planning [7].

Despite increases in the number of studies on the relationship between the neighborhood environment and health, the potential impact of the neighborhood environment across a range of health outcomes has not been fully explored. For instance, existing studies have focused on specific CVD risk factors such as obesity [7–9], metabolic syndrome [10], physical activity [11,12] and walking [13]. In addition, a recent study reviewed obesity-related outcomes [14]. Although Mayne et al. 2015[14] used quasi-experiment in their review, the study centered on obesity and related risk factors. Previously, the association between built environment and obesity has received wide publication. However, no study has broadly reviewed the relationship of neighborhood environment with major CVD outcomes and risk factors, while such a review is necessary to guide future research and policy formulation in this sector [15]. Therefore, the purpose of this study is to synthesize the studies on the association between a number of neighborhood environment attributes and CVD risks.

## Methodology

### Data sources/ search strategy

A comprehensive search was conducted to identify all research articles published from 2005 to 2015 that examine neighborhood environment, major CVD outcomes and selected risk factors (Table 1). English language articles were identified from the following databases: EBSCO (including: Academic Search, CINAHL, Global Health, Health Source: Nursing/academic and Medline) and Science Direct. Significant studies were identified using any of the following keywords: neighbourhood environment, perceived neighborhood environment, perceived built environment, land use mix diversity, physical activity, social environment, overweight or obesity, hypertension, diabetes mellitus, metabolic syndrome, coronary heart disease and myocardial infarction.

**Table 1:** Database Search strategies.

CINAHL	
**No**	**Search terms**
01	Neighborhood environment
02	Physical activity
03	Adults
04	#1 and #2 and #3
**Master File Premier**	
01	Built environment
02	Overweight or obesity
03	Adults
04	#1 and #2 and #3
**Science Direct**	
01	Perceived built environment
02	Diabetes mellitus
03	Adults
04	#1 and #2 and #3
**EBSCO host** (including; academic search complete, CINAHL, Global health, Health source: nursing/academic, Medline)	
01	Perceived neighborhood environment
02	Hypertension
03	Adult
04	#1 and #2 and #3
05	Perceived built environment
06	Diabetes mellitus
07	Adults
08	#5 and #6 and #7
09	Land use mix diversity
10	Metabolic syndrome
11	Adults
12	#9 and #10 and #11
13	Social environment
14	Myocardial infarction
15	adults
16	#13 and #14 and #15
17	Perceived neighborhood environment
18	Coronary heart disease
19	adults
20	#17 and #18 and #adults

### Study selection

**Fig 1:**
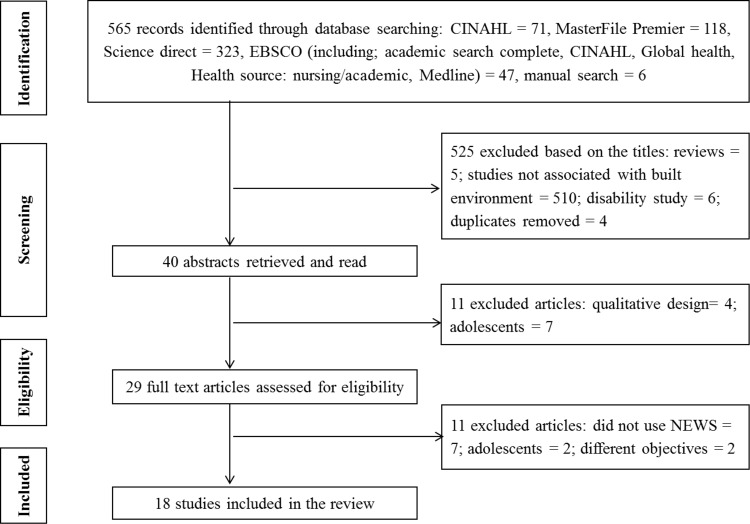
Flow Chart of included studies. This figure represents the flow of the literature review conducted according to the Preferred Reporting Items for Systematic Reviews and Meta-Analyses (PRISMA) guidelines [16].

Titles and abstracts of all identified articles were assessed for their potential eligibility. Full texts of potentially eligible articles were then retrieved and their eligibility was verified against the study eligibility criteria. Fig 1 (a flow chart of included studies; see appendix) represents the flow of the literature review conducted according to the Preferred Reporting Items for Systematic Reviews and Meta-Analyses (PRISMA) guidelines [16], S1 Table (PRISMA 2009 checklist). Studies published in English were included if: 1) they used a Geographic Information System (GIS) [17] or subjectively assessed one or more of the built environment factors categorized according to the validated and reliably tested ‘Neighborhood Environment Walkability Scale’ (NEWS) which is a better questionnaire to assess the local environment [18]; 2) examined the relationship with any of the major CVD outcomes including myocardial infarction, coronary heart disease and stroke; 3) examined selected risk factors including physical activity (categorized in domains were considered), overweight or obesity, hypertension and diabetes mellitus; 4) were original reports on studies conducted among subjects aged 18 years and above; and 5) if the purpose of the studies were to explore the association between the variables of interest using multivariate analyses. Exclusion criteria were as follows: 1) Studies exclusively conducted on adolescents; 2) studies that employed a qualitative design; 3) systematic review papers; 4) publications from studies where subjects had difficulty with walking and 5) studies that did not meet the criteria for current review.

### Data extraction

The information extracted included the first authors’ name, publication year, the sample size, gender, age range of the subjects, country and city where the study was conducted, study design, study tool (assess neighborhood environment), exposure assessment (any of the neighborhood environment attributes), outcome assessment (CVD outcomes or risk factors), and measures of association. Data abstraction, classification, and quality assessment of each study were conducted by two reviewers independently. A third reviewer was consulted if there was disagreement.

### Quality appraisal of the studies

In order to assess the methodological quality for each study selected, the ‘Strengthening the reporting of observational studies in epidemiology’ (STROBE) checklist [19] was adapted in accordance with the objectives of this study. For instance, this included the: sample size, setting, design, study tool (assessing neighborhood environment), exposure, outcome measure and association according to the area of this study. The final PRISMA checklist included I8 items that assessed the quality of this study. Each item scored one point if full reporting was met, or zero if not or partially reported.

### Data synthesis

Due to differences in research questions, exposure measurements, outcome measurements and methods across studies, a formal meta-analysis was not possible. Thus, the current review applied a semi-quantitative procedure [7]. The aim of this semi-quantitative procedure was to allow a rapid assessment of the strength of the evidence of an association between the exposure and the outcomes of interest by reducing a range of results from heterogeneous analytical designs to two binary questions [20]: a) did the study under review show a positive or negative association between the built environmental attributes and the outcome of interest? b) and, if so, was this finding statistically significant (p<0.05)? Hence, estimates of associations between neighborhood environment attributes, CVD risk factors and major outcomes were extracted from the eligible studies according to their substantive relevance and methodological findings and results summarized (Table 2). However, to take into account potential publication bias, we did not limit our analysis on papers published in peer-reviewed journals. References of finally included records were additionally checked. Built environment studies assessing relationship with CVD risks and outcomes are relatively recent. Therefore, this study restricted the search for a specific time period and database. Contrary, no quantitative assessment for risk of bias in individual studies was performed. However, in each study sample size, number of observations per built environment and total number of considered CVD risks and outcomes were checked, because small sample sizes result in biased effect estimates.

## Results

### Overview of the study selection process

An overview of the types of the articles selected is provided in Table 2, highlighting the author, country, gender, age, sample size, study design, study tools (assess neighborhood environment), exposure measures, outcome measures and their associations. The electronic search yielded 565 articles from the selected databases; MasterFile Premier = 118, CINAHL = 71, Science Direct = 323, EBSCO (including; Academic Search, CINAHL, Global Health, Health Source: Nursing/academic, and Medline) = 47, manual search = 6. After title/abstract screening, 525 articles were excluded for not meeting inclusion criteria. Of the excluded articles, 510 articles were unrelated to neighborhood environmental attributes, CVD risk and CVD outcomes, 5 were systematic reviews, 6 were conducted in a population with clinical conditions (disability), and another 4 were duplicates. The abstracts of 40 citations were then obtained and retrieved. Out of these abstracts, 11 were excluded since 4 were qualitative design and 7 were conducted among adolescents. Thus, 29 full text articles were assessed for eligibility. Of these, 11 were excluded as 7 did not use NEWS, 2 were conducted among adolescents and another 2 did not meet the objective of the review to measure BE (S2 Table, excluded articles). Therefore only 18 articles were finally eligible for inclusion in the current review. The flow chart in Fig 1 shows the process leading to the number of included articles for the review.

**Table 2:** Studies that have assessed neighborhood environmental attributes and CVD* risk factors and outcomes.

Research Methodology										
Author (s)/year	Country	Setting	Gender	Age	Sample	Study design	Tool	Exposure measure	Outcome measure	Association
Adams et al., 2012[23]	USA	Urban	F	66–77	368 (Baltimore)	C-S	NEWS	Land-use mix-diversity, access to services, infrastructure for walking/cycling, aesthetics, traffic safety, and crime safety	PA	Neighborhood attributes differed by as much as 10 minutes/day for moderate-to-vigorous PA, 1.1 hours/week for walking, and 50 minutes/week for leisure PA (p ≤ 0.001).
					360 (Seattle)				BMI	BMI was lower in the high walkable/recreational dense neighborhoods (p ≤ 0.001).
Witten et al., 2012[24]	New Zealand	Urban	F/M	20–65	2033	C-S	GIS	Residential density, street connectivity, land use mix	PA	1-SD increases in destination access, street connectivity, and dwelling density were associated with self-reported transport, leisure, or walking PA, with increased odds ranging from 21% (street connectivity with leisure PA, 95%-CI: 0%, 47%) to 44% (destination accessibility with walking, 95% CI: 17%, 79%).
Hanibuchi et al. 2011[35]	Japan	Urban/suburban/rural	F/M	65+	9414	C-S	GIS	Residential density, street connectivity, number of local destinations, access to recreational spaces, and land slope	Leisure time, sports activity and total walking time	Population density and presence of parks or green spaces had positive associations with PA
Heesch et al. 2014[29]	Australia	Urban	F/M	40–45	11036	C-S	NEWS-A	Traffic volume, aesthetics, and crime, recreational facilities, traffic slowing device, cul-de-sacs, four-way intersections, hilly streets	Cycling	Perceived environmental attributes were positively associated with cycling (p < 0.05).
Wilson et al. 2011[30]	Australia	Urban	F/M	40–65	10286	C-S	GIS	Public transport, shop, and park street lights, river or coast connectivity, residential density, hilliness, tree coverage, bikeways, and network distance to nearest river or coast, public transport, shop, and park.	Total Minutes walking in the past week	Walking was positively associated with connectivity, residential density, least tree coverage, bikeways and streetlights.
Martinez et al.,2012[25]	USA	Community	F/M	18–65	672	C-S	NEWS	Neighborhood Safety (heavy traffic, crime, stray dogs, street lights and crosswalks), socio support	LTPA	Neighborhood attributes were negatively associated with meeting LTPA guidelines.
Zhou et al. 2013[37]	China	Schools	F/M	40+	478	C-S	NEWS-A	Residential density, diversity of land use, facility access, street connectivity, walking and cycling facilities, aesthetics, pedestrian safety, and crime safety	PA	Participants from downtown areas were more likely to engage in transportation related PA and leisure-time PA than respondents living in the suburbs. Residential density was positively associated with recreational or leisure-based PA. Street connectivity was negatively associated with leisure time PA. Moderate vigorous PA was negatively associated with traffic safety. Environmental attributes were not significantly associated with transportation PA.
Atkinson et al. 2005[21]	USA	Urban	F/M	20–65	102	C-S	NEWS	Land-use mix-diversity, access to services, infrastructure for walking/cycling, aesthetics, traffic, safety, and crime safety	PA	Environment attributes were significantly associated with both vigorous-intensity self-reported and objectively measured physical activity. The vigorous and total activity accelerometer measures were correlated with street connectivity.
Pruchno et al. 2014[36]	USA	Community	F/M	50–74	5688	Survey	GIS	Supermarkets, grocery stores, local convenience stores, and fast-food restaurants	BMI	High densities of fast-food restaurants were positively associated with obesity. Supermarkets were not associated with obesity.
Drewnowski et al. 2012[26]	USA	Urban	F	50–79	60775	Survey	GIS	Density of grocery store and supermarkets and fast food restaurants (1.5 miles)	Blood Pressure	High densities of stores/supermarkets were associated with low diastolic blood pressure.
Li et al. 2009[31]	USA	Urban	F/M	50–75	1145	C-S	GIS	Land use mix, street connectivity, number of public transit stations, and amount of green and open spaces. Density of fast-food restaurants	Blood Pressure	High walkable neighborhoods were associated with decreased systolic and diastolic blood pressure. Neighborhoods of low walkability but with high density of fast-food restaurants were significantly associated with BP. The negative effect of fast-food restaurants on blood pressure was attenuated in high-walkable neighborhoods.
Baldock et al. 2012[27]	Australia	Suburban	F/M	18 +	1324	C-S	NEWS-AU	Land-use mix-diversity, access to services, infrastructure for walking/cycling, aesthetics, traffic safety, and crime safety	Metabolic syndrome	Metabolic syndrome was negatively associated with local land-use mix, positive aesthetics, and infrastructure for walking, and was positively associated with perceived crime and barriers to walking
Müller-Riemenschneider et al. 2013[34]	Australia	Rural	F/M	25 +	5970	C-S	GIS	Residential density, street connectivity, land use mix	Metabolic syndrome.	High walkable neighborhoods were associated with low obesity and type-2 diabetes mellitus, but not with hypertension
Coffee et al. 2013[32]	Australia	Urban	F/M	18 +	3593	C-S	GIS	Walkability, index-dwelling density, intersection density, land-use mix and retail footprint	Metabolic syndrome	High walkability neighborhoods were associated with lower cardiometabolic risk.
Sundquist et al. 2014[33]	Sweden	Urban	F/M	18 +	512061	Survey	GIS	Residential density, street connectivity, land use mix	Type 2 diabetes	Walkability was negatively associated with type 2 diabetes
Kan et al. 2008[38]	USA	Communities	F/M	45–64	13309	Survey	GIS	Traffic density/distance to major roads	CHD	High traffic density was positively associated with CHD
Hamano et al. 2013[28]	Sweden	Urban	F/M	35–80	4319674	Longitudinal	GIS	Fast food restaurant, bars/pubs, PA and healthcare facilities	Stroke	High density fast food restaurants and pubs/bars were positively associated with stroke. Physical activity and healthcare facilities were negatively associated with stroke
Chum & O’Campo 2015[22]	Canada	Community	F/M	25+	2411	C-S	GIS	Violent crimes, environmental noise, and proximity to a major road, food, stores, parks/recreation, fast food restaurants	MI, angina, CHD, stroke, and CHF	High crime rate, environmental noise, and proximity to a major road were positively associated with increased CVDs. Reduced access to food stores, parks/recreation, and increased access to fast food restaurants were associated with increased CVDs.

*CVD, cardiovascular disease; F, female; M, male; CS, cross-section; NEWS-AU, neighborhood environment walkability scale-Australia; PA, physical activity; BMI, body mass index; GIS, geographic information system; LTPA, leisure time physical activity; MI, myocardial infarction; CHD, Coronary heart disease; CHF; coronary heart failure.

### General characteristics of the studies included

Table 2 depicts the descriptive characteristics of the included studies. The year of study ranged between 2005 [21] and 2015 [22], with 27.8% (n = 5) being published in 2012 [23–27]. Sample sizes varied across studies, ranging from 102 [21] to 4,319,674 [28]. In all, 55.5% (n = 10) of the studies were conducted in urban [21,23,24,26,28–32,33] areas as compared to rural [34], suburban [27] and urban/suburban/rural [35]. Community based studies [22,25,33,36] constituted 22.2% (n = 4) compared to one institution based study [37]. The reported ages of the participants ranged from 18 [25,27,32,33] to 80 years [28]. Most studies included females and males [21, 22,24,25,27–36] (88.9%; n = 16) with only 11.1% (n = 2) being in females only [23,26]. Sixteen studies (88.9%) were conducted in high-income countries [21–33,34, 36,38], 11.1% (n = 2) in middle income countries [35,37] and 38.9% (n = 7) were conducted in the USA alone [21,23,25,26,31,33,36]. Of all included studies, 94.4% (n = 17) were cross-sectional [21–22,26,27,29–38] with one being longitudinal [28].

### CVD risk factors and outcomes covered across studies

Of the 18 studies reviewed, 44.4% focused on physical activity [21,23–25,29,30,35,37], 16.7% on body mass index [23,35], 5.6% on blood pressure [26], 5.6% on diabetes mellitus [33] and 16.7% on metabolic syndrome [27,34,32]. Furthermore, 16.7% of studies [22,28,38] focused on coronary heart disease, stroke and heart failure, Table 2.

### Measurement of neighborhood environmental attributes

The majority of the studies (66.7%) used GIS [22,24,26,28,30–36] to assess neighbourhood environment attributes, while 33.3% used NEWS questionnaires [21,23,25,27,29,34] (Table 2**)**.

### Association between neighborhood environment attributes and CVD risk

The majority of the reported associations of neighborhood environmental attributes with CVD risk factors and outcomes were statistically significant (p < 0.05) with effects estimates in the expected direction, and only two studies with mixed results, comparing neighborhood environmental attributes with transport related physical activity [37] and hypertension [34] respectively, reported no significant association, Table 2. Forty four percent of studies [21,23–25,29,30,35,37] reported variety of neighborhood environmental attributes associated with physical activity domains. Conversely, 11.1% of studies reported neighborhood environmental attributes were associated with body mass index [23,36] and blood pressure [26,31]. In addition, 16.6% studies reported metabolic syndrome [27, 32,34] and only one study indicated diabetes mellitus [33] to be related with Built environment attributes. Similarly, 16.6% of studies showed a significant association between neighborhood environmental attributes and myocardial infarction, coronary heart disease, congestive heart failure, angina and stroke [22, 28,38], Table 2.

## Discussion

This review has shown that a variety of neighborhood environmental attributes are associated with physical activity. Furthermore, density of fast food restaurants, supermarkets/grocery stores and high walkable neighborhood environments were associated with body mass index, blood pressure, diabetes mellitus and metabolic syndrome. In addition, high density traffic, road proximity and high density of fast food restaurants were associated with major CVD outcomes.

Our results are consistent with other studies [11,39]. In particular, physical activity was associated with safe footpaths and recreational facilities [40,41] and walking [42]. The results indicate that urban attributes such as street connectivity, residential density, recreational facilities and availability of traffic devices improves neighborhood walkability which may promote walking, leisure and transport related to physical activity which, consequently, lowers the incidence of CVDs. For instance, environmental attributes are thought to increase active transportation and lessen the need for private automobile use to accomplish daily tasks, which, in turn, lowers body mass index [43].

This review found that neighborhood environmental attributes such as fast-food restaurants and high walkable neighborhood environment were associated, either positively or negatively with body mass index, blood pressure and metabolic syndrome risk. Previous studies have reported similar results on the association between food environment and BMI [41,44,45] or blood pressure [10]. Greater accessibility to fast food restaurants may encourage people to make food choices at odds with ‘healthy’ dietary recommendations by making these choices easier [46]. Another explanation is that limited access to supermarkets may incentivize visits to convenience stores or fast food restaurants outlets [47] thereby increasing the chance of consuming unhealthy foods, with consequential increases in individual body mass indices and blood pressure levels.

Living in high walkable neighborhoods was associated with a lower prevalence of high body mass index, diabetes mellitus and metabolic syndrome risk. Similar results have been reported elsewhere [10]. Neighbourhood environmental attributes may increase an individual’s active transportation related to the physical activity needed to accomplish daily tasks and thus lower the [43]. For example, a higher population density may support increased recreational opportunities and supermarkets offering a better supply of healthy foods, and so explaining associations between body mass index [48] and metabolic syndrome risk [10]. Moreover, high walkable neighborhood environments are associated with promoting recreational and transport related physical activity [49], participation in which eventually assists in lowering the prevalence of obesity or metabolic syndrome risks. Furthermore, an increase in intersection density in the neighborhood may promote walking through providing more route options and may regulate traffic [48].

Our study also observed that major CVD outcomes are related to built environment attributes. Specifically, a study has reported similar results on proximity to traffic [50]. Environmental attributes include proximity to stores, and access to supermarkets and non-fast food stores which may, consequently, affect the extent to which individuals walk and the food choices they make, which governs their diet and thus links to CVDs [51, 52]. Likewise, high traffic volumes have been associated with noise and air pollution which are linked to major CVDs. In addition, road proximity has been linked with low individual and neighborhood socioeconomic status, both of which have been shown to be associated with CVDs [53].

### Limitations of the review

One limitation of this study is the paucity of primary research on the association between neighborhood environmental attributes and CVD risk and major CVDs in an African context. Almost all publications included in the review were cross-sectional, thus causal inferences in the relationships could not be determined. The exclusion of studies not conducted in English also detracts from this study. In addition, this study reviewed few CVD risk factors with selected CVDs. Furthermore, we did not perform meta-analysis to derive pooled estimates of the association across studies. This was due to the much heterogeneity in measures of associations used across included studies, as well as the wide range of outcomes examined across studies. Future studies should explore any association between CVDs and other environmental attributes such as tobacco use, alcohol use and air pollution in order to have a broader understanding of other moderating effects. To our knowledge, this is the first review to document the associations between both objectively and subjectively measured built environment attributes and selected CVD risk and major CVDs. Methods of classification and categorization of the findings in this study follow those of other similar studies, facilitating comparisons. Moreover, this study further contributes to illustrating that studies from developed countries use comparable methodologies to studies from less well developed countries, such as this one.

### Conclusion

This study shows that both objective and perceived neighborhood environmental attributes are linked to CVD and its risk factors. The information gathered here from studies that explored neighborhood environmental attributes and their association with CVD risks and major CVD outcomes will help guide policy makers on the neighborhood environmental, transportation, health and education to improve intervention programs by local government and for people at a ‘grass-roots’ level. Future studies should further explore the associations of CVD risk and CVD outcomes with a broad set of neighborhood attributes using a longitudinal approach to better understand the direction of effects.

## Supporting Information

S1 Table PRISMA 2009-checklist. (DOC)

S2 Table Excluded full articles from the review. (DOCX)
