# Supplementary material for: Built Environment, Selected Risk Factors and Major Cardiovascular Disease Outcomes: A Systematic Review
Source: PLoS One. 2016 Nov 23;11(11):e0166846. doi: 10.1371/journal.pone.0166846 (PMC5120821; doi:10.1371/journal.pone.0166846)
Supplement: S2 Table — (DOCX) [file pone.0166846.s002.docx]

**S2 table. Excluded full articles from the review**

| **Item #** | **Authors** | **Title** | **Reason (s) for exclusion** |
| --- | --- | --- | --- |
| 1 | Nathan *et al*., 2012 [1] | The association between neighborhood greenness and cardiovascular disease: an observational study | The authors used Normalized Difference Vegetation Index (NDVI) instead of NEWS |
| 2 | Kim *et al*., 2014 [2] | Perceived neighborhood social cohesion and myocardial infarction | Perceived social environment variables was not part of the NEWS |
| 3 | Cunningham-Myrie *et al.,* 2014 [3] | Associations between neighborhood effects and physical activity, obesity, and diabetes: The Jamaica Health and Lifestyle Survey2008 | The age group did not meet the criteria for the review |
| 4 | Piccolo *et al.,* 2014 [4] | The role of neighborhood characteristics in racial/ethnic disparities in type 2 diabetes: Results from the Boston Area Community Health (BACH) Survey | Objective of the study did not met the criteria for the review |
| 5 | Hirsch *et al.,* 2014 [5] | Change in Walking and Body Mass Index Following Residential Relocation: The Multi-Ethnic Study of Atherosclerosis | Walk Score index was used instead of NEWS |
| 6 | Fish *et al.,* 2010 [6] | Association of Perceived Neighborhood Safety on Body Mass Index | A survey question was not adopted from NEWS |
| 7 | James *et al.,* 2013 [7] | Urban Sprawl, Physical Activity, and Body Mass Index:Nurses’ Health Study and Nurses’ Health Study II | Sprawl index was used instead of the News |
| 8 | Drewnowski *et al.,* 2012 [8] | Obesity and Supermarket Access: Proximity or Price? | The characteristics of the independent variable (supermarkets) did not meet the criteria for the review |
| 9 | Casagrande *et al.,* 2011[9] | Association of Walkability With Obesity in Baltimore City, Maryland | The Pedestrian Environment Data Scan (PEDS)/ was a measure instead of the NEWS |
| 10 | Oyeyemi *et al.,* 2011[10] | Perceived environmental correlates of physical activity and walking in African young adults | The age group did not meet the criteria for the review |
| 11 | Powell-Willey *et al*., 2013 [11] | Relationship between Perceptions about Neighborhood Environment and Prevalent Obesity: Data from the Dallas Heart Study | Survey question not adopted from the NEWS |

NEWS, neighborhood environment walkability scale

**Reference**

1. Nathan A, Pereira G, Foster S, Hooper P, Saarloos D, Giles-Corti B. Access to commercial destinations within the neighbourhood and walking among Australian older adults. Int J Behav Nutr Phys Act. 2012; 9(1):133. doi:10.1186/1471-2458-12-466.

2. Kim ES, Hawes a. M, Smith J. Perceived neighbourhood social cohesion and myocardial infarction. J Epidemiol Community Heal. 2014; 68(11):1020-1026

3. Cunningham-Myrie CA, Theall KP, Younger NO, Mabile EA, Tulloch-Reid MK, Francis DK, et al. Associations between neighborhood effects and physical activity, obesity, and diabetes: The Jamaica Health and Lifestyle Survey 2008. J Clin Epidemiol. 2015; 68(9):970–8.

4. Piccolo RS, Duncan DT, Pearce N, McKinlay JB. The role of neighborhood characteristics in racial/ethnic disparities in type 2 diabetes: Results from the Boston Area Community Health (BACH) Survey. Soc Sci Med. 2015;130:79–90. doi.org/10.1016/j.socscimed.2015.01.041.

5. Hirsch JA, Roux AVD, Moore KA, Evenson KR, Rodriguez DA. Change in walking and body mass index following residential relocation: The multi-ethnic study of atherosclerosis. Am J Public Health. 2014; 104(3):49–57.

6. Fish JS, Ettner S, Ang A, Brown AF. Association of perceived neighborhood safety on body mass index. Am J Public Health. 2010; 100(11):2296–303.

7. James P, Troped PJ, Hart JE, Joshu CE, Colditz GA, Brownson RC, et al. Urban sprawl, physical activity, and body mass index: Nurses’ health study and nurses' health study II. Am J Public Health. 2013; 103(2):369–75.

8. Drewnowski A, Aggarwal A, Hurvitz PM, Monsivais P, Moudon A V. Obesity and supermarket access: Proximity or price? Am J Public Health. 2012; 102(8):74–81.

9. Casagrande SS, Gittelsohn J, Zonderman AB, Evans MK, Gary-Webb TL. Association of walkability with obesity in Baltimore City, Maryland. Am J Public Health. 2011; 101(SUPPL. 1):318–25.

10. Oyeyemi AL, Adegoke BO, Sallis JF, Oyeyemi AY, De Bourdeaudhuij I. Perceived crime and traffic safety is related to physical activity among adults in Nigeria. BMC Public Health. 2012; 12(1):294.

11. Powell-Wiley TM., Ayers CR., de Lemos JA., Lakoski SG., Vega GL., Grundy S., Das SR., Banks-Richard K. and AM. Relationship between Perceptions about Neighborhood Environment and Prevalent Obesity: Data from the Dallas Heart Study. Obes (Silver Spring). 2009; 27(1):E14–21.
